# External validation of molecular subtype classifications of colorectal cancer based on microsatellite instability, CIMP, BRAF and KRAS

**DOI:** 10.1186/s12885-019-5842-7

**Published:** 2019-07-11

**Authors:** Elizabeth Alwers, Hendrik Bläker, Viola Walter, Lina Jansen, Matthias Kloor, Alexander Arnold, Julia Sieber-Frank, Esther Herpel, Katrin E. Tagscherer, Wilfried Roth, Jenny Chang-Claude, Hermann Brenner, Michael Hoffmeister

**Affiliations:** 1 Division of Clinical Epidemiology and Aging Research, German Cancer Research Center (DKFZ), Im Neuenheimer Feld 581, 69120 Heidelberg, Germany; 2 Department of General Pathology, Institute of Pathology, Charité University Medicine Hospital, Berlin, Germany; 3 Department of Applied Tumor Biology, Institute of Pathology, University of Heidelberg, Heidelberg, Germany; 4 Institute of Pathology, University Hospital Heidelberg, Heidelberg, Germany; 5 NCT Tissue Bank, National Center for Tumor Diseases (NCT), Heidelberg, Germany; 6 Institute of Pathology, University Medical Center Mainz, Mainz, Germany; 7 Division of Cancer Epidemiology, German Cancer Research Center (DKFZ), Heidelberg, Germany; 8 Genetic Tumor Epidemiology Group, University Medical Center Hamburg-Eppendorf, Hamburg, Germany; 9 Division of Preventive Oncology, German Cancer Research Center (DKFZ) and National Center for Tumor Diseases (NCT), Heidelberg, Germany; 10 German Cancer Consortium (DKTK), German Cancer Research Center (DKFZ), Heidelberg, Germany

**Keywords:** Colorectal cancer, Molecular subtypes, Cancer-specific survival, External validation

## Abstract

**Background:**

Competing molecular classification systems have been proposed to complement the TNM staging system for a better prediction of survival in colorectal cancer (CRC). However, validation studies are so far lacking. The aim of this study was to validate and extend previously published molecular classifications of CRC in a large independent cohort of CRC patients.

**Methods:**

CRC patients were recruited into a population-based cohort study (DACHS). Molecular subtypes were categorized based on three previously published classifications. Cox-proportional hazard models, based on the same set of patients and using the same confounders as reported by the original studies, were used to determine overall, cancer-specific, or relapse-free survival for each subtype. Hazard ratios and confidence intervals, as well as Kaplan-Meier plots were compared to those reported by the original studies.

**Results:**

We observed similar patterns of worse survival for the microsatellite stable (MSS)/BRAF-mutated and MSS/KRAS-mutated subtypes in our validation analyses, which were included in two of the validated classifications. Of the two MSI subtypes, one defined by additional presence of CIMP-high and BRAF-mutation and the other by tumors negative for CIMP, BRAF and KRAS-mutations, we could not confirm associations with better prognosis as suggested by one of the classifications. For two of the published classifications, we were able to provide results for additional subgroups not included in the original studies (men, other disease stages, other locations).

**Conclusions:**

External validation of three previously proposed classifications confirmed findings of worse survival for CRC patients with MSS subtypes and BRAF or KRAS mutations. Regarding MSI subtypes, other patient characteristics such as stage of the tumor, may influence the potential survival benefit. Further integration of methylation, genetic, and immunological information is needed to develop and validate a comprehensive classification that will have relevance for use in clinical practice.

## Background

Colorectal cancer (CRC) is a leading cause of cancer incidence and mortality worldwide [1, 2]. Patient survival is generally well predicted according to the TNM stage; however, there is still stage independent variability due to the molecular heterogeneity of the tumor [3]. Molecular characteristics of the tumor that also affect patient survival, such as MSI, BRAF and KRAS, have only recently been included in the 8th edition of the UICC recommendations as additional indicators for clinical practice guidance.

A deficiency in the MMR system, represented by the microsatellite instability (MSI-H) phenotype, can be found in approximately 15% of CRC cases, and is associated with both sporadic (~ 12%) and hereditary (~ 3%) cancers. Patients with the MSI-high phenotype seem to have better survival than patients with microsatellite stable (MSS) tumors [4, 5]. The MSI status is closely associated with other characteristics of the tumor, such as BRAF mutations, early stage, proximal location, and higher degree of immune infiltration. Some clinical trials focusing on stage III patients receiving chemotherapy have reported conflicting results regarding the prognosis of MSI-high patients [6–8], which could be explained by the varying tumor characteristics. Mutations in BRAF V600E and KRAS exon 2 were also associated with worse patient prognosis in previous studies; however, these associations seem to be restricted to MSS tumors [9, 10]. In combination with the TNM stage information, KRAS mutations and the MSI status of the tumor are used in clinical practice to determine treatment options [11, 12].

According to UICC recommendations, before a modified staging system can be endorsed for use in clinical practice, a classification must be validated in various external cohorts using different patient populations in several settings [13]. In a previous systematic review, multiple studies that proposed molecular subtype classifications of CRC (including markers such as MSI, BRAF and KRAS) and determining their associations with patient survival were identified [14]. No classification found significant associations for all proposed subtypes and only two performed external validation of their results [15, 16]. Given the importance of external validation of such classifications, the aim of this study was to validate CRC molecular subtype classifications previously published in the literature and extend their application to additional patient subgroups not previously investigated, using an independent cohort of patients from a population-based study.

## Methods

### Study population and design

The DACHS study is a population-based case-control study conducted in the Rhine-Neckar region in southern Germany, in which incident CRC cases are followed-up as a cohort. Details of the study design have been reported previously [17]. Briefly, patients over 30 years of age, with a histologically confirmed diagnosis of CRC, and able to participate in an interview in German, were recruited between 2003 and 2010 from 22 hospitals in the region. Patients with available tumor tissue samples, molecular characterization on MSI, BRAF, KRAS and CIMP, and follow-up were included in this analysis.

Baseline characteristics, and medical and family history of CRC were collected by trained interviewers at the time of diagnosis using a standardized questionnaire. Tumor characteristics and stage of disease (6th edition of the TNM staging manual) were obtained from medical records and pathology reports. Follow-up assessment at 3 and 5 years after diagnosis included information on the type of treatment, comorbidities, cancer recurrence, and vital status. Vital status and date of death were obtained from population registries, and determination of cause of death was based on death certificates obtained from the pertinent health authorities.

### Molecular marker determination

Tumor tissue analyses were performed on formalin fixed, paraffin embedded (FFPE) samples. As previously described, MSI status was determined using a mononucleotide marker panel (BAT25, BAT26 and CAT25) [18]. BRAF V600E was determined independently by two experienced pathologists (HBl, MK) using IHC analyses in tissue microarray blocks (52%) and by mutational analysis (48%) using Sanger sequencing (exon 15). No significant differences were observed in the proportion of aberrant cases identified by the two techniques. KRAS mutations were determined using DNA samples by the single stranded conformational polymorphism technique (48%) or by Sanger sequencing (52%) (exon 2). CIMP status was defined using a five marker methylation panel (MLH1, MINT1, MINT2, MINT31 and MGMT) and classified according to the number of hyper-methylated loci: CIMP negative (none), CIMP-low (1 or 2 loci), or CIMP-high (3 or more loci).

### Studies for comparison

Studies with proposed molecular subtypes of colorectal or colon cancers that provided an estimation of the association with survival for each subtype were identified in a previous systematic review [14] and used as a base to perform this external validation analysis. Studies were included in the systematic review if they suggested a classification system based on at least three markers and reported results for survival by each subtype. In this validation analysis we were able to include classifications of CRC based on the molecular subtypes previously suggested by Jass [19], including MSI and CIMP status, and mutation status of BRAF and KRAS genes [16, 20, 21]. Table 1 presents a summary of the population and study characteristics of the studies included in this analysis.

**Table 1:** Characteristics of studies for validation

	Studies with proposed classifications to be validated			Validation set
	Samadder et al. [20] 2013	Phipps et al. [21] 2015	Sinicrope et al. [16] 2015	
Study	Iowa Women’s Health Study	Seattle Colon Cancer Family Registry	Phase III randomized trial NCCTG N0147	DACHS study
Study design	Cohort	Population-based cohort	Cohort from clinical trial	Population-based cohort
N	370	1189	2720	1915
Country	US	US	US	Germany
Population	Women aged 55–69 resident in Iowa	Persons aged 20–74 resident in Washington state and women 50–74 in postmenopausal study	Patients aged 19–86 with resected stage III colon carcinomas in Alabama region	Patients aged 30–96 resident in Rhine-Neckar region
Cancer	Colorectal	Colorectal	Colon	Colorectal
Stage	I - IV	I - IV	III	I - IV
Recruitment time	1986–2002	1998–2007	2004–2012	2003–2010
Treatment	Any	Any	FOLFOX or FOLFOX+cetuximab	Any
Outcomes	CSS, OS	CSS, OS	DFS	CSS, RFS, OS
Median follow-up	NR	NR	4.9 years	5.3 years

*DACHS* Darmkrebs, Chancen der Verhütung durch Screening, *US* United States, *CSS* cancer-specific survival, *DFS* disease-free survival, *OS* overall survival, *NR* not reported

### Validation and statistical analysis

Demographic and clinico-pathological characteristics were analyzed for the entire study population using descriptive statistics. Patients were categorized into the same subtypes proposed by the three studies. Cox-proportional hazards regression models were used to calculate cancer-specific (CSS), relapse-free (RFS), or overall survival (OS) for each subtype. CSS was defined as time from diagnosis until death from CRC, RFS until reappearance of disease, metastases or death from CRC, and OS until death from any cause. All models were adjusted using the same set of variables reported by the original studies. Classifications that were developed only among women, or only in stage III colon cancer patients were validated in both the selected sub-population and the entire patient cohort, to investigate whether the classification would yield similar results in an unselected patient cohort.

For the validation of the proposed classifications, the hazard ratios reported by the original studies were compared with those obtained in the DACHS cohort after creating subtypes equal to those proposed by the original studies. As suggested by Royston and Altman [22], Kaplan-Meier plots were created to evaluate the discrimination between subtypes and to allow a visual comparison of discrimination with the originally published plots. Additionally, Uno’s c-statistic was calculated for each of the models [23]. This method provides a measure of discrimination that accounts for time-to-event data and censoring in survival analyses [24]. All statistical analyses were performed in SAS version 9.4 (*SAS Institute Inc., Cary, NC, USA*).

## Results

### DACHS population characteristics

Among 1915 cases with complete tumor marker data, patients were classified according to the TNM system as stage I in 355 (19%), stage II in 644 (34%), stage III in 652 (34%), and stage IV in 264 (14%) cases. The MSI and CIMP status, and mutations in BRAF were significantly associated with age, sex, location and stage of disease in descriptive analyses (Table 2). During a median follow-up of 5.3 years, 548 (29%) patients experienced disease recurrence, and 624 (33%) patients died, 414 (66%) of whom from CRC. CRC-specific deaths occurred in 20 (9%) MSI-high cases, 44 (28%) BRAF mutated cases, 144 (23%) KRAS mutated cases, and 66 (19%) CIMP-high cases.

**Table 2:** Descriptive characteristics of CRC patients according to molecular markers in the DACHS cohort

Variable n (%)	Overall *n = 1915*	MSS *n = 1698* *(89%)*	MSI *n = 217* *(11%)*	p-val	BRAF wt *n = 1759* *(92%)*	BRAF mut *n = 156* *(8%)*	p-val	KRAS wt *n = 1293* *(68%)*	KRAS mut *n = 622* *(32%)*	p-val	CIMP-low *n = 1572* *(82%)*	CIMP-high *n = 343* *(18%)*	p-val
Age groups													
< 65	629 (33)	566 (33)	63 (29)	*< 0.001*	598 (34)	31 (20)	*< 0.001*	437 (34)	192 (31)	*0.425*	551 (35)	78 (23)	*< 0.001*
65–74	654 (34)	596 (35)	58 (27)		609 (35)	45 (29)		433 (33)	221 (36)		542 (35)	112 (33)	
> 75	632 (33)	536 (32)	96 (44)		552 (31)	80 (51)		423 (33)	209 (34)		479 (31)	153 (45)	
Sex													
Female	808 (42)	690 (41)	118 (54)	*< 0.001*	705 (40)	103 (66)	*< 0.001*	547 (42)	261 (42)	*0.887*	625 (40)	183 (53)	*< 0.001*
Male	1107 (58)	1008 (59)	99 (46)		1054 (60)	53 (34)		746 (58)	361 (58)		947 (60)	160 (47)	
Location													
Proximal colon	684 (36)	501 (30)	183 (85)	*< 0.001*	555 (32)	129 (83)	*< 0.001*	441 (35)	243 (40)	*0.100*	464 (30)	220 (64)	*< 0.001*
Distal colon	497 (26)	476 (28)	21 (10)		483 (28)	14 (9)		344 (27)	153 (25)		441 (28)	56 (16)	
Rectum	714 (38)	703 (42)	11 (5)		702 (40)	12 (8)		495 (39)	219 (36)		648 (42)	66 (19)	
TNM stage													
Stage I	355 (19)	321 (19)	34 (16)	*< 0.001*	336 (19)	19 (12)	*0.068*	239 (19)	116 (19)	*0.276*	293 (19)	62 (18)	*0.015*
Stage II	644 (34)	525 (31)	119 (55)		580 (33)	64 (41)		453 (35)	191 (31)		504 (32)	140 (41)	
Stage III	652 (34)	594 (35)	58 (27)		603 (34)	49 (31)		428 (33)	224 (36)		553 (35)	99 (29)	
Stage IV	264 (14)	258 (15)	6 (3)		240 (14)	24 (15)		173 (13)	91 (15)		222 (14)	42 (12)	
Chemotherapy													
No	1045 (55)	893 (53)	152 (71)	*< 0.001*	948 (54)	97 (63)	*0.036*	714 (56)	331 (53)	*0.343*	829 (53)	216 (63)	*< 0.001*
Yes	857 (45)	794 (47)	63 (29)		800 (46)	57 (37)		568 (44)	289 (47)		731 (47)	126 (37)	

*MSS* microsatellite stability, *MSI* microsatellite instability, *BRAF wt* BRAF wild type, *BRAF mut* BRAF mutated, *KRAS wt* KRAS wild-type, *KRAS mut* KRAS mutated

*p*-values from Chi-square or Fischer’s exact tests for the association of the two variables

### External validation results

Table 3 presents the molecular classification, original results, and validation results obtained with the DACHS cohort. In general, the distribution of patients from the DACHS study within each classification was similar to the one reported by the original studies. Larger differences between the original studies and our study were observed for the ‘Traditional’ (45.9% vs 56.8%) and ‘Alternate’ (38.4% vs 32.0%) subtypes proposed by Samadder et al. [20], and the ‘Type 4’ subtype proposed by Phipps et al. [21] (4.6% vs 1.7%). Due to the selective recruitment of stage III patients in the study by Sinicrope et al. [16], numbers of patients were lower for all subtypes in the DACHS study.

**Table 3:** Comparison of patient survival according to the identified classifications with external validation and additional subgroups in the DACHS cohort

						Original studies				DACHS study			
Author / Population	Subtypes	MSI	BRAF	KRAS	CIMP	n	(%)	HR (95%CI)	HR (95%CI)	n	(%)	HR^a^ (95%CI)	HR^a^ (95%CI)
Samadder et al. [20] 2013								CSS	OS			CSS	OS
Women, CRC	Traditional	–	–	–	–	170	45.9	1	1	366	56.8	1	1
	Serrated	any	+	–	+	58	15.7	1.56 (0.9–2.8)	1.23 (0.8–1.8)	72	11.2	1.37 (0.7–2.5)	1.04 (0.6–1.7)
	Alternate	–	–	+	–	142	38.4	1.26 (0.7–2.4)	0.85 (0.6–1.3)	206	32	1.09 (0.7–1.6)	0.93 (0.7–1.3)
	Total					370				644			
Men	Traditional	–	–	–	–			*Not reported in original study*		598	65.5	1	1
	Serrated	any	+	–	+					32	3.5	0.89 (0.3–2.5)	0.95 (0.4–2.0)
	Alternate	–	–	+	–					283	31	1.21 (0.9–1.7)	1.24 (1.0–1.6)
	Total									913			
Both sexes	Traditional	–	–	–	–			*Not reported in original study*		964	61.9	1	1
	Serrated	any	+	–	+					104	6.7	1.35 (0.8–2.2)	1.09 (0.7–1.6)
	Alternate	–	–	+	–					489	31.4	1.15 (0.9–1.5)	1.08 (0.9–1.3)
	Total									1557			
Phipps et al. [21] 2015								CSS	OS			CSS	OS
All stages, CRC	Type 1	+	+	–	+	100	8.4	0.54 (0.3–1.0)	1.05 (0.8–1.4)	77	4.8	0.93 (0.5–1.8)	0.83 (0.5–1.4)
	Type 2	–	+	–	+	55	4.6	1.84 (1.2–2.8)	1.40 (1.0–2.0)	27	1.7	1.83 (1.0–3.4)	1.55 (0.9–2.7)
	Type 3	–	–	+	–	353	29.7	1.25 (1.0–1.5)	1.23 (1.0–1.5)	489	30.4	1.18 (0.9–1.5)	1.13 (0.9–1.4)
	Type 4	–	–	–	–	631	53.1	1	1	964	60	1	1
	Type 5	+	–	–	–	50	4.2	0.42 (0.2–0.9)	0.74 (0.5–1.2)	50	3.1	1.04 (0.4–2.5)	1.51 (0.8–2.7)
	Total					1189				1607			
Sinicrope et al. [16] 2015								DFS ^b^				RFS	
Stage III, colon	Non BRAF/KRAS	–	–	–		1331	48.9	1		137	49.1	1	
Chemotherapy	Mut KRAS	–	–	+		945	34.7	1.48 (1.3–1.7)		102	36.6	1.39 (0.8–2.4)	
N0147 trial	Mut BRAF	–	+	–		189	6.9	1.43 (1.1–1.8)		12	4.3	2.77 (1.1–7.2)	
	dMMR sporadic	+	+	any	MLH1	184	6.8	1.10 (0.8–1.5)		16	5.7	1.15 (0.4–3.2)	
	dMMR familial	+	–	any		71	2.6	0.77 (0.5–1.3)		12	4.3	0.33 (0.1–2.6)	
	Total					2720				279			
All stages, colon	Non BRAF/KRAS	–	–	–				*Not reported in original study*		578	51.3	1	
	Mut KRAS	–	–	+						361	32.1	1.31 (1.0–1.7)	
	Mut BRAF	–	+	–						53	4.7	3.01 (1.9–4.7)	
	dMMR sporadic	+	+	any	MLH1					84	7.5	0.60 (0.3–1.1)	
	dMMR familial	+	–	any						50	4.4	0.36 (0.1–1.0)	
	Total									1126			
All stages, CRC	Non BRAF/KRAS	–	–	–				*Not reported in original study*		1055	57.3	1	
	Mut KRAS	–	–	+						578	31.4	1.18 (1.0–1.4)	
	Mut BRAF	–	+	–						63	3.4	2.96 (2.0–4.4)	
	dMMR sporadic	+	+	any	MLH1					84	4.6	0.54 (0.3–1.0)	
	dMMR familial	+	–	any						60	3.3	0.58 (0.3–1.2)	
	Total									1840			

*MSI* microsatellite instability, *dMMR* deficient mismatch-repair, *HR* hazard ratio, *CSS* cancer-specific survival, *OS* overall survival, *DFS* disease-free survival, *RFS* relapse-free survival

^a^Adjusted for what the original study adjusted for: Samadder et al.: Age, location, grade, stage, chemotherapy, radiotherapy. Phipps et al.: Age, sex, stage, BMI, year of diagnosis, smoking. Sinicrope et al.: Age, sex, location, T stage, N stage, grade, lymph nodes examined, chemotherapy

^b^DFS: Defined as “time from date of randomization to first documented disease recurrence or death (due to all causes), whichever occurred first”. Approximated here with RFS

In a study restricted to female CRC patients, Samadder et al. [20] proposed three molecular subtypes and two additional ‘unassigned’ subgroups. The two unassigned groups were not clearly defined, and therefore not included in this validation. Both BRAF and KRAS mutated subtypes showed worse CSS compared to the all-negative baseline, however none of the associations were statistically significant. In the DACHS cohort, the magnitudes and directions of the measures of effect were similar in all subtypes for CSS and OS, but no significant associations were observed either. The c-statistic for the CSS and OS models was 0.828 and 0.780, respectively.

Phipps et al. [21] proposed five molecular subtypes and found significantly worse CSS for the MSS subtypes (types 2 and 3), and better CSS for the type 5 (MSI-high, BRAF non-mutated, KRAS non-mutated, CIMP-negative) subtype in comparison to the type 4 (MSS, BRAF non-mutated, KRAS non-mutated, CIMP-negative) subtype [21]. In the DACHS cohort, the magnitude and direction of the effect for CSS and OS were similar for the MSS subtypes (types 2 and 3). The MSI-high subtypes were not significantly associated with better survival (type 1: HR = 0.93 [0.5–1.8]; type 5: HR = 1.04 [0.4–2.5]), regardless of the BRAF or KRAS mutational status. The c-statistic for the CSS and OS models was 0.797 and 0.744, respectively.

Sinicrope et al. [16] described five molecular subtypes in a cohort of stage III colon cancer patients recruited in the N0147 clinical trial. Significantly worse CSS was observed for both KRAS mutated and BRAF mutated MSS subtypes, and no significant associations were found for either of the MSI-high subtypes compared to the MSS non-mutated subtype [16]. These findings were similar in the DACHS cohort, where HRs for RFS showed worse survival in the MSS groups and no significant associations in the MSI-high subtypes. The c-statistic for this model was 0.723.

### Extended analyses in patient subgroups not available from the original studies

In exploratory analyses, the classifications were validated using additional subgroups of DACHS patients (**see** Table 3). Similar to the results in the main analysis, no significant associations were found for the classification proposed by Samadder et al. [20] in analyses restricted to men, although a tendency towards worse CSS and OS in the MSS, KRAS mutated subgroup was observed. This tendency was also observed for the subgroup analyses including both sexes, where HRs for CSS were similar to those reported for women by the original study. Stage-specific analyses for the classification proposed by Phipps et al. [21] suggested a better survival for MSI subtypes only in early stage patients (data not shown). Extended analyses of Sinicrope’s [16] classification including patients with all stages of colon and colorectal cancers showed similar associations for the MSS subtypes, and a borderline significant association of the MSI subgroups with RFS.

### Visual assessment of agreement with original studies

**Fig. 1:**
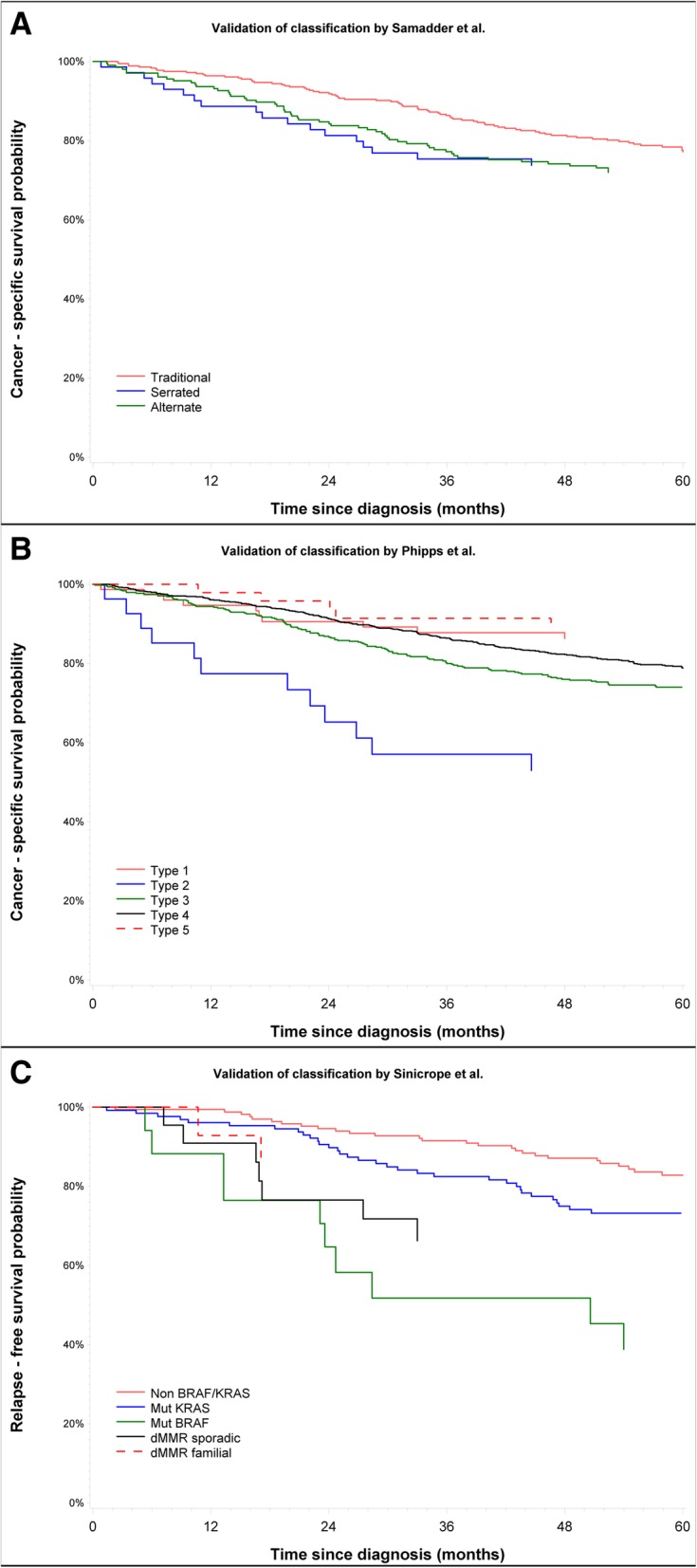
Kaplan-Meier curves for each classification within DACHS patient cohort. A. Cancer-specific survival for the classification by Samadder et al. [20] B. Cancer-specific survival for the classification by Phipps et al. [21] C. Relapse-free survival for the classification by Sinicrope et al. [16]

Figure 1 presents Kaplan-Meier plots for survival in the DACHS cohort after categorizing patients according to each of the proposed classifications. In the DACHS cohort (Fig. 1a), the Kaplan-Meier plot suggested a better survival for the ‘Traditional’ pathway curve compared to the one published by Samadder et al. [20] (Fig. 3B in the original study). No differences were observed between the ‘Alternate’ and ‘Serrated’ pathways. For the classification proposed by Phipps et al. [21], a visual comparison with the originally published Kaplan-Meier plots allowed to infer good agreement with the type 2 subtype, which corresponds to MSS, BRAF mutated, CIMP-positive tumors. The other subtypes in this classification also showed similar patterns of survival (Fig. 1b). However, type 1 and type 5 (MSI-high subtypes) showed better survival compared to the all-negative type 4 subtype in the original study (Fig. 1 in the original study [21]). The survival curves based on the classification by Sinicrope et al. [16] (Fig. 1c), showed a worse pattern of RFS for the MSS/BRAF mutated subtype. Even though the outcomes were different (DFS and RFS) the patterns observed in the Kaplan-Meier plots were similar to the ones published by the authors in their own external validation cohort (Fig. 3 in the original study [16]).

## Discussion

In this external validation study of previously proposed molecular subtype classifications for the prediction of survival among CRC patients, we found that MSS cancers with BRAF or KRAS mutations generally conferred a worse prognosis compared to tumors with no such mutations. This finding was reported by two previous studies, and we were able to validate their results both in similar patient populations and additional subgroups that were not included in the original studies. Overall, all subtypes of the three proposed classifications showed similar hazard ratios and levels of significance compared to the ones reported by the original studies, except for two MSI-high subtypes proposed by Phipps et al. [21] for which we found no statistically significant associations of better survival.

In their study, Phipps et al. [21] described that patients with MSI-high tumors with or without mutations in BRAF or KRAS (types 1 and 5) had better survival than those with MSS tumors. This reflects the findings of several previous studies and meta-analyses where MSI-high tumors had a better prognosis than MSS tumors [5]. In our study, we found better survival for MSI-high tumors compared to MSS tumors but no statistically significant associations for the specific MSI subtypes (1 and 5). We attribute this difference to the different way in which patients were distributed among the subtypes: i) the proportion of stage I patients differs between the studies for type 1 (47% in original study vs 13% in our study) and type 5 (50% in original study vs 12% in our study); ii) Phipps et al. [21] included a large proportion of patients from a cohort of postmenopausal women and a second recruitment round of patients diagnosed before 50 years of age, whereas patients in our study reflect an older patient population with equal distribution of men and women. These differences in population characteristics might also be responsible for the observed survival in the different subtypes.

None of the other studies included here provided an estimate of survival for MSI-high subtypes with no mutations in BRAF and KRAS. For example, Samadder et al. [20] provided only one subtype where MSI status could be either negative or positive, and found no significant associations with survival. Sinicrope et al. [16] described two MSI-high subtypes without specifying the KRAS mutation status and found no significant associations with DFS. Both subtypes were also not associated with RFS in the validation analyses. This underlines the need to not only discriminate the MSI status of a tumor in any classification [3], but to also include information on the BRAF and KRAS mutational status. When conducting the analyses in different sets of patients (e.g. including all stages and locations) the findings were similar.

Only one of the three studies (Sinicrope et al. [16]) had performed a validation analysis in an external patient population. No measures of effect were provided for the validation cohort; however, the Kaplan-Meier plot created in our validation analysis showed similar patterns to the one provided in this study, especially for the BRAF mutated subtype. Survival curves for the other two studies also showed good agreement with the ones generated in our analysis, particularly the MSS/BRAF mutated subtype described by Phipps et al. [21].

Other CRC classifications have been published in recent years [15, 25–27]. The consensus molecular subtype (CMS) group included gene expression information in their classification, and proposed four CRC subtypes for which prediction of survival was only significant for one [25]. The CMS classification system was not included in the present validation analysis, because some information required for the definition of the CMS subtypes was not available from our study. The CMS1 subtype, however, which corresponds to the type 1 tumors reported by Phipps et al. [21], was not significantly associated with better survival, similar to our validation results for type 1 tumors. Even though the proposed CMS subtypes were derived from a complex methodology including information from several international studies, the information required to classify a patient into the subtypes may not be readily available in every clinical practice.

Other studies provided analyses stratifying for the MSS status of the tumor, instead of including MSS as a part of the classification and thus, were not included in this validation analysis [9, 28]. These studies included stage III colon cancer patients recruited in clinical trials for chemotherapy treatment and found significant associations only for MSS tumors with time to relapse and survival after relapse [9, 28]. A recent analysis showed that including molecular information as well as clinical and pathological characteristics in survival models improved their ability to predict overall survival in stage II and III colon cancer patients [29]. This study represents an important first step in optimizing the existing prognostic classification system of CRC and will allow for additional efforts to achieve an ideal classification. Additionally, the Immunoscore showed independent prognostic value for CRC survival after adjusting for the TNM stage and shows promising ability to complement the current system [30–32]. These results might be influenced by the close relation between immune infiltration and the MSI status of a tumor [33, 34], but could add value to a prognostic classification that is to complement the traditional staging system. All these diverse studies reflect the increasing international interest in the development of a more comprehensive classification that could help clinicians provide a more personalized treatment to CRC patients [35].

Due to the exploratory character of newly proposed molecular CRC classification systems, validation studies in external patient cohorts are essential before the usefulness of the proposed classification can be judged. In this large cohort study, we were able to perform validation of three previously proposed classification systems by incorporating the same set of molecular markers. We attempted to imitate the original analyses by adjusting for the same set of confounders in each case; however, there might still be differences in the assessment and definition of the variables leading to differences in the calculated estimates. On the other hand, not all hitherto proposed classifications could be validated in our study [14], since not all information required to construct the molecular subtypes was available. Also, although it is a large study, sample size decreases when stratifying patients into several subtypes and restricting the populations to match the ones reported by the original studies, which limits the power for the statistical analyses. Finally, the time periods when the patients were recruited span different decades and this could mean the chemotherapy regimens used were different between the studies.

## Conclusions

In conclusion, the results of this validation analysis contribute to the evolving interest in the development of an extended clinically meaningful classification for CRC. None of the published classifications has so far provided a definitive subtyping that allows to predict patient survival in all groups. Our results support the conclusion that MSS subtypes including BRAF or KRAS mutations have a worse prognosis compared to those without the latter mutations and that those subtypes can be readily generalized to most other patient populations. The role of CIMP status was less clear in the prediction of survival, and it is known to be highly associated with MSI. Our extended analysis supported that the observed associations were similar in patient subgroups that were not included in the original studies. However, as for the MSI subtypes, where other characteristics such as stage, location and sex are highly correlated, may require more careful evaluation before generalizing their potential survival benefit. Also, further information from methylation, gene expression, and immune response analyses in tumor tissue may help to further improve the definition of clinically relevant molecular subtypes. The present knowledge about molecular subtypes of colorectal cancer suggests that the stage of disease remains the most important predictor of survival, and that more research is needed to find molecular tumor markers or combinations that help to complement this system.

## Data Availability

The datasets used and/or analyzed during the current study are not publicly available due to data protection laws but are available from the corresponding author on reasonable request.

## Abbreviations

CIMP: CpG island methylator phenotype
CRC: Colorectal cancer
CSS: Cancer-specific survival
DACHS: Darmkrebs: Chancen der Verhütung durch Screening
DFS: Disease-free survival
EGFR: Epidermal growth factor receptor
IHC: Immunohistochemistry
MMR: Mismatch repair
MSI: Microsatellite instability
MSS: Microsatellite stable
OS: Overall survival
RFS: Relapse-free survival
UICC: Union for International Cancer Control
